# Supplementary material for: Autoimmunity gene IRGM suppresses cGAS‐STING and RIG‐I‐MAVS signaling to control interferon response
Source: EMBO Rep. 2020 Jul 27;21(9):e50051. doi: 10.15252/embr.202050051 (PMC7507369; doi:10.15252/embr.202050051)
Supplement: Supplementary file 1 — Appendix [file EMBR-21-e50051-s001.pdf]

Title: Autoimmunity Gene IRGM Suppresses cGAS-STING and RIG-I-MAVS Signaling to control Interferon Response

Table of content:

Content	Page Number
Appendix Figure S1	2
Appendix Figure S2	3
Appendix Figure S3	4
Appendix Figure S4	5
Appendix Figure S5	6
Appendix Table S1	7-8

Appendix Figure S1

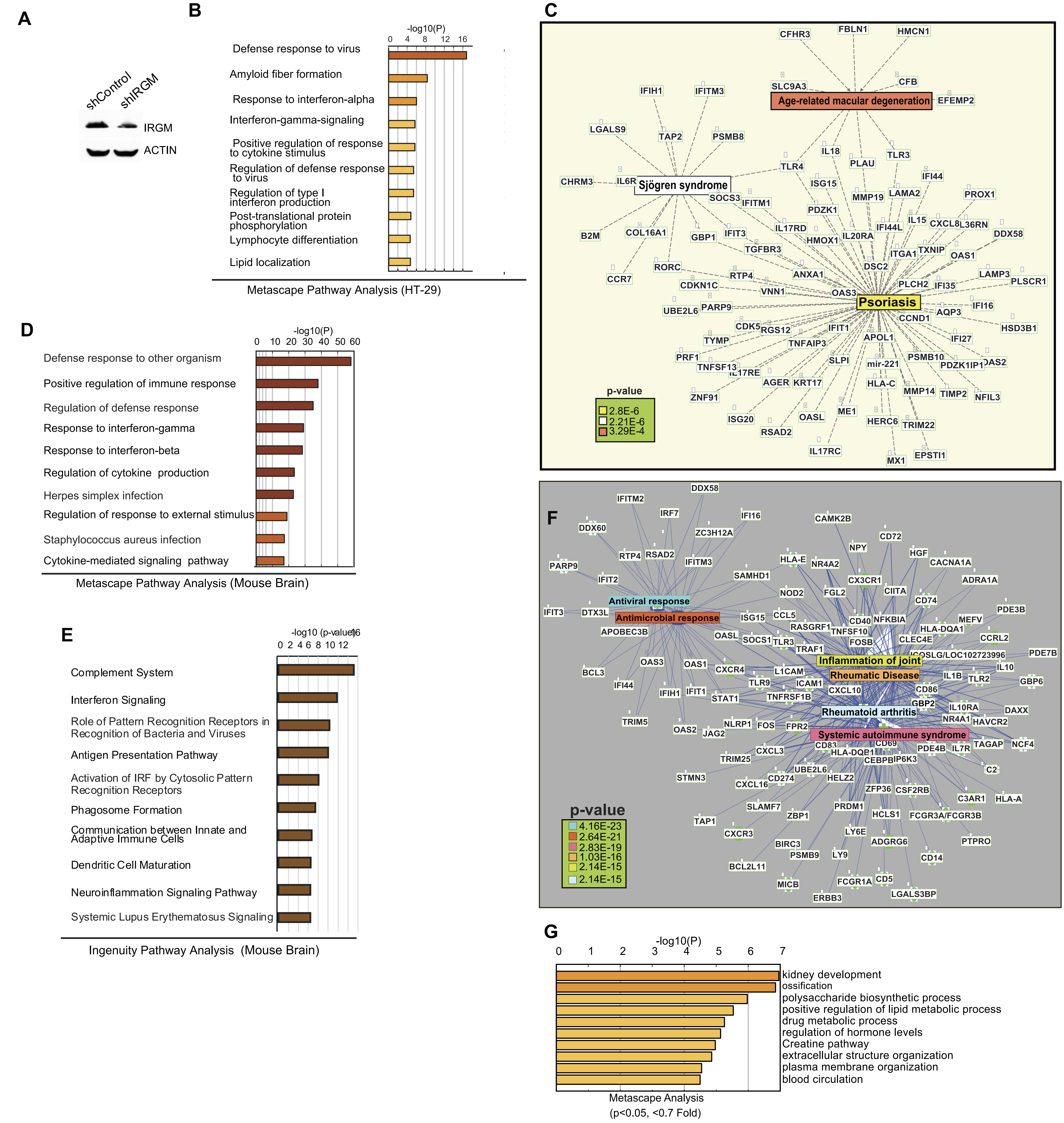

Appendix Figure S1. RNA-seq data depicts that IRGM controls anti-viral and autoimmunity signaling pathways.
(A) IRGM knockdown efficiency analyzed by western blot using cell lysate of HT29 stably expressing control shRNA or IRGM shRNA.
(B) Metascape pathway analysis using sets of genes induced (1.5 fold, p<0.05, (Wald Test), n=3 biological replicates) in IRGM shRNA knockdown HT29 cells compared to control shRNA cells.
(C) Network pathway analysis using IPA. The molecular network of genes connected with diseases associated with genes (1.5 fold, p<0.05 (Wald Test), n=3 mice) upregulated in Irgm1-/- mice brain. The complete list is documented in Dataset EV2.
(D) Metascape pathway analysis using sets of genes induced (1.5 fold, p<0.05 (Wald Test), n=3 mice) in the brain of Irgm1-/- knockout mice compared to Irgm1+/+ wild type mice.
(E) Ingenuity pathway analysis using sets of genes induced (1.5 fold, p<0.05 (Wald Test), n=3 mice) in the brain of Irgm1-/- knockout mice compared to Irgm1+/+ wild type mice.
(F) Network pathway analysis using IPA. The molecular network of genes connected with diseases/function associated with genes (1.5 fold, p<0.05 (Wald Test), n=3 mice) upregulated in Irgm1 knockout mice BMDM's. The complete list is documented in Dataset EV2.
(G) Bar graph represents top canonical biological pathways upregulated in GO-based metascape pathway analysis using a set of genes repressed (≤ 0.7 fold, p<0.05 (Wald Test), n=3 mice) in Irgm1 knock-out mice BMDM's compared to wild type mice BMDM's.

.

Appendix Figure S2

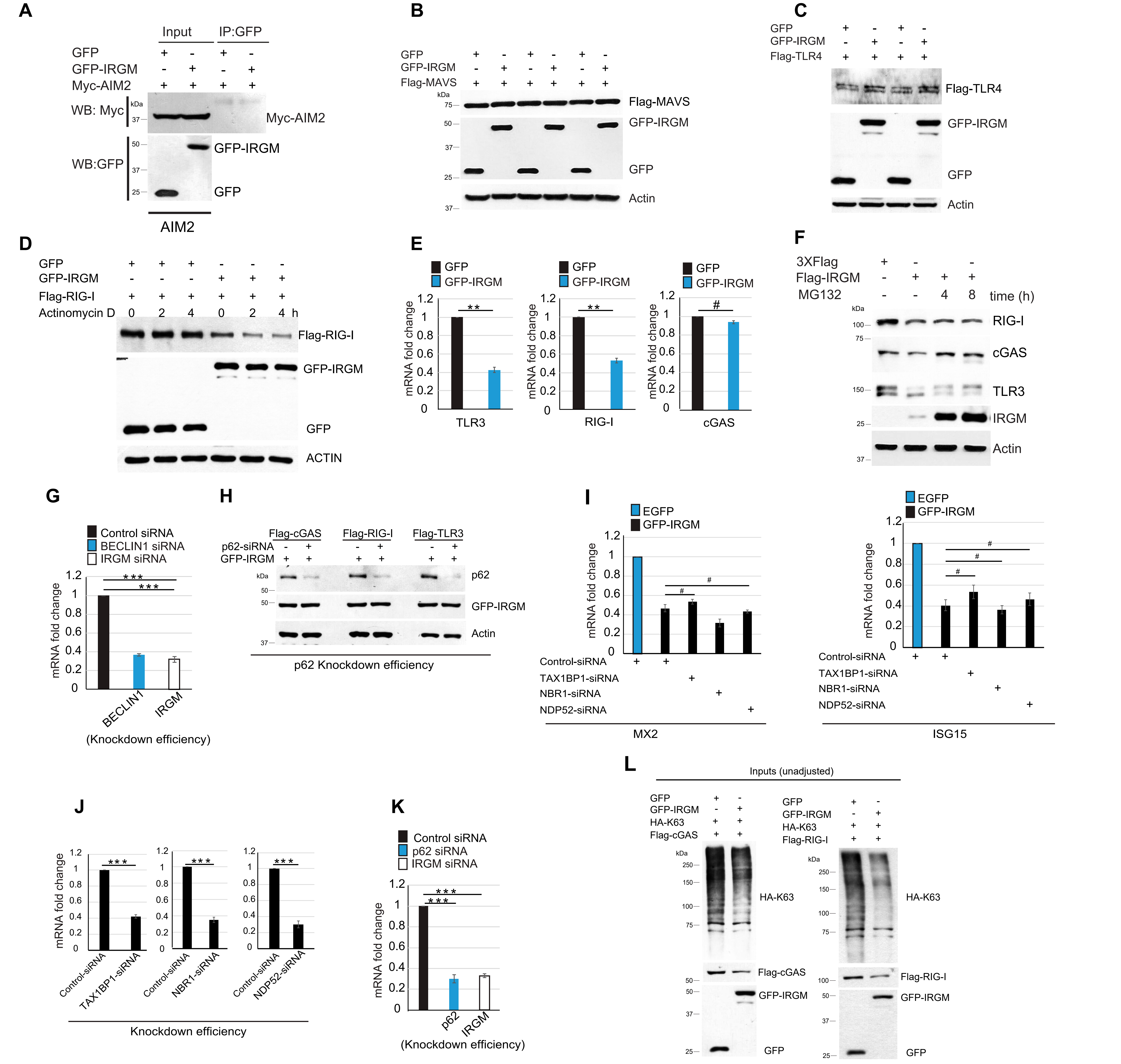

Appendix Figure S2. IRGM interacts and degrades cytoplasmic DNA and RNA sensor proteins to constrain IFN response.
(A) Co-immunoprecipitation (Co-IP) analysis of interaction between GFP-IRGM and Myc-AIM2 in HEK293T cell lysates.
(B) Western blot analysis with lysates of HEK293T cells transiently expressing GFP or GFP-IRGM and Flag-MAVS. (n=3 technical repeats shown)
(C) Western blot analysis with lysates of HEK293T cells transiently expressing GFP or GFP-IRGM and Flag-TLR4. (n=2 technical repeats shown).
(D) Western blot analysis with lysdates of HEK293T cells transiently expressing GFP or GFP-IRGM and Flag RIG-I and treated with Actinomycin-D (5μg/ml, 0 h, 2 h and 4 h).
(E) qRT-PCR analysis with total RNA isolated from THP-1 cells trasiently transfected with GFP and GFP-IRGM as indicated. Mean ± SD, n = 3 (biological replicates), **$p < 0.005$, Student's unpaired t-test. #p = Non significant.
(F) Control and HT29 cells stably expressing Flag IRGM were treated with MG132 (20 μM, 4 h and 8 h) and were subjected to immunoblotting with the indicated antibodies.
(G) qRT-PCR analysis with total RNA isolated from THP-1 cells transfected with control siRNA or BECLIN1 siRNA or IRGM siRNA as indicated. Mean ± SD, n = 3 (biological replicates), ***$p < 0.0005$, Student's unpaired t-test.
(H) Western blot analysis of control and p62 siRNA transfected HEK-293T cell lysates transiently expressing GFP-IRGM and probed with indicated antibodies.
(I) qRT-PCR analysis with RNA isolated from control siRNA, TAX1BP1 siRNA, NBR1 siRNA or NDP52 siRNA transfected THP-1 cells expressing EGFP or GFP-IRGM as indicated. Mean ± SD, n = 3 (biological replicates), #p = Non significant, Student's unpaired t-test.
(J) qRT-PCR analysis with RNA isolated from control siRNA, TAX1BP1 siRNA, NBR1 siRNA or NDP52 siRNA transfected THP-1 cells. Mean ± SD, n = 3 (biological replicates), ***$p < 0.0005$, Student's unpaired t-test.
(K) qRT-PCR analysis with total RNA isolated from THP-1 cells transfected with control siRNA or p62 siRNA or IRGM siRNA as indicated. Mean ± SD, n = 3 (biological replicates), ***$p < 0.0005$, Student's unpaired t-test.
(L) Western blot analysis of Input samples (Unadjusted-1:1 ratio) from Co-immunoprecipitation (Co-IP) experiment of the ubiquitination of Flag-cGAS or Flag-RIG-I in the presence of EGFP or GFP-IRGM and probed with indicated antibodies.

Appendix Figure S3

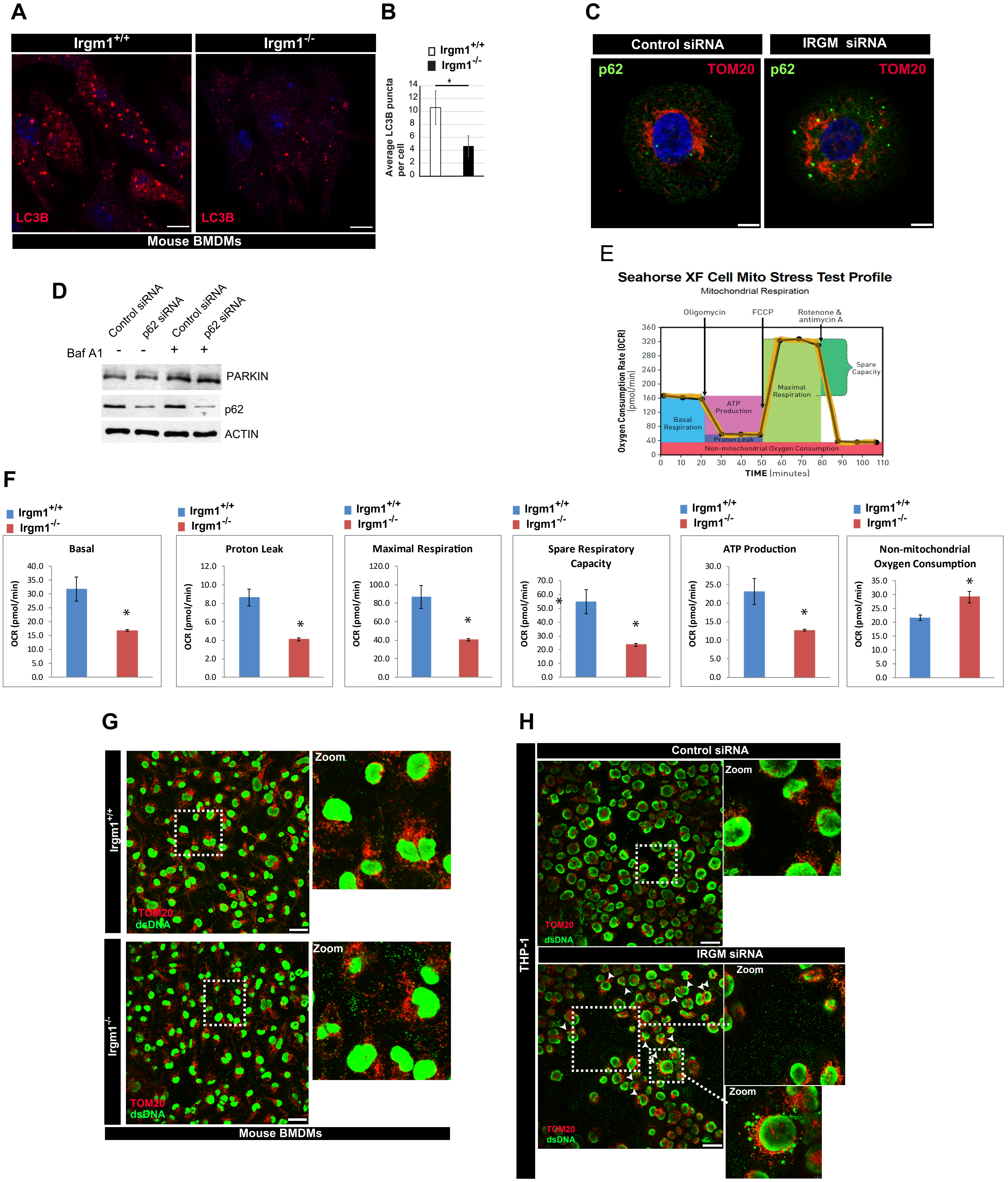

Appendix Figure S3. IRGM depletion results in defective mitophagy and enhanced cytosolic DAMPs.
(A) Representative confocal images of Irgm1+/+ and Irgm1-/- mice BMDMs immunostained with LC3B (red) antibody. The panels show LC3B puncta. Scale bar, 10 µm.
(B) Bar diagram showing average LC3B puncta in Irgm1+/+ and Irgm1-/- mice BMDMs. (n = 3 (biological replicates), mean ± SD, *p ≤ 0.05, Student's unpaired t-test).
(C) Representative confocal images of control siRNA and p62 siRNA transfected THP-1 cells, immunostained with p62 (green) and TOM20 (red) antibodies. Scale bar, 3 µm.
(D) Western blot analysis of control siRNA and IRGM siRNA transfected THP-1 cells untreated or treated with Bafilomycin A1 (300 nM, 4 h), probed with indicated antibodies.
(E-F) Seahorse XF Cell mito stress test for analysis of mitochondrial function in Irgm1+/+ and Irgm1-/- mice BMDM cells. The experiments were performed using the XF24 extracellular flux analyzer. (C) The standard Seahorse XF Cell mito stress test profile and, (D) The contribution of associated parameters including basal oxygen consumption, proton leak, maximal oxygen consumption, spare respiration capacity, ATP-linked oxygen consumption, and non-mitochondrial oxygen consumption was plotted, respectively. Results shown represent mean ± standard error (n = 3 (biological replicates), mean ± SD, *p ≤ 0.05, Student's unpaired t-test).
(G) Representative confocal images of Irgm1+/+ and Irgm1-/- mice BMDMs immunostained with dsDNA (green) and TOM20 (red) antibodies. The Irgm1- zoom panels show extracellular DNA in cytoplasm.Scale bar, 25 µm. Zoom panels are digital magnifications.
(H) Representative confocal images of control siRNA and IRGM siRNA transfected THP-1 cells immunostained with dsDNA (green) and TOM20 (red) antibodies. The zoom panels show extracellular DNA and cytoplasmic micronuclei. The white arrows in IRGM siRNA panel depict the micronuclei. Scale bar, 25 µm. Zoom panels are digital magnifications.

Appendix Figure S4

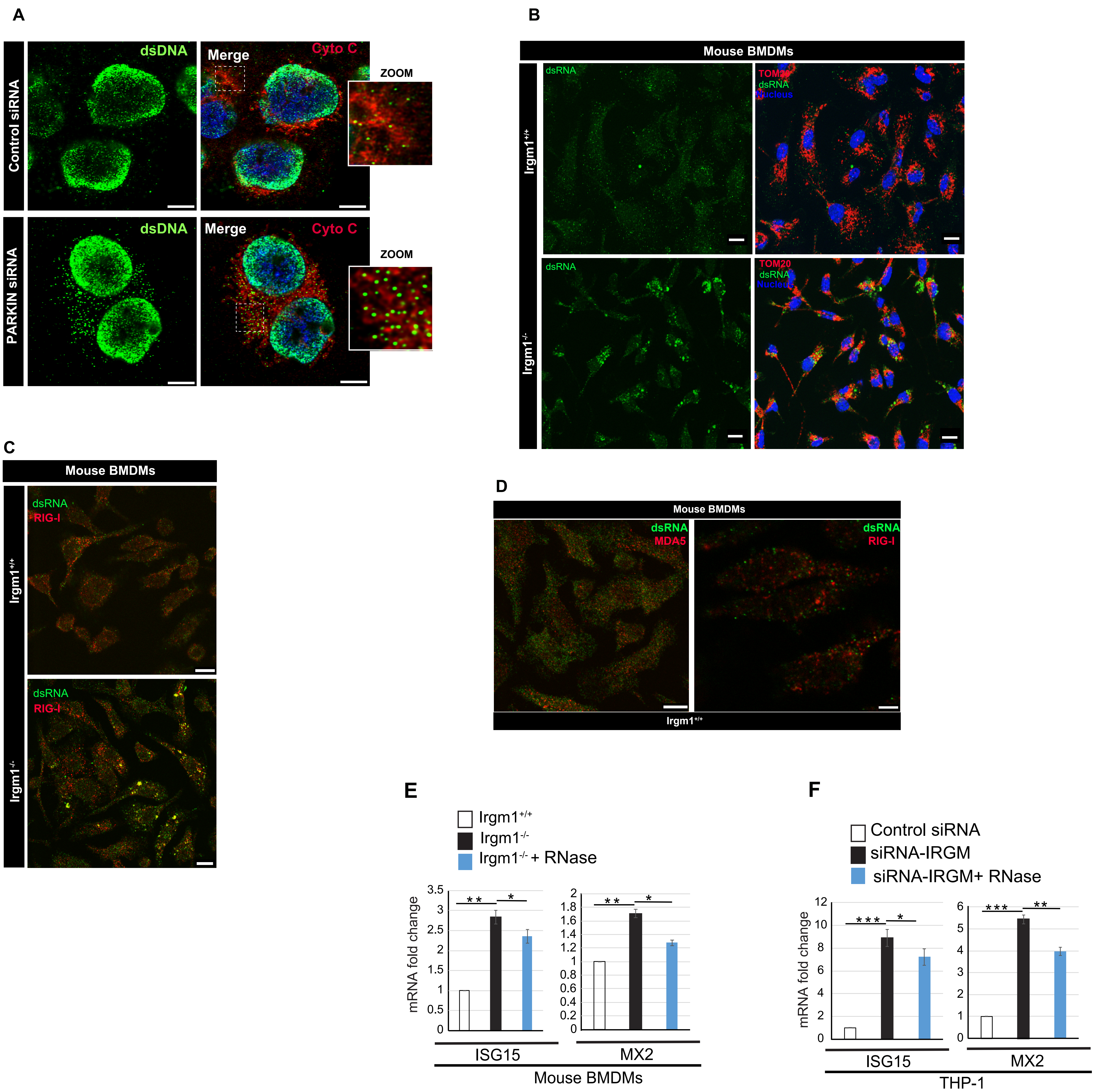

Appendix Figure S4. Cytosolic DAMPs in IRGM-depleted cells invoke nucleic acid-sensing pathway for activation of IFN response.
(A) Representative confocal images of control siRNA and PARKIN siRNA transfected THP-1 cells immunostained with dsDNA (green) and Cytochrome C (red) antibodies. The zoom panels show cytosolic dsDNA. Scale bar, 5 µm. Zoom panels are digital magnifications.
(B) Representative confocal images of Irgm1+/+ and Irgm1-/- mice BMDMs immunostained with dsRNA (green) and TOM20 (red) antibodies.Scale bar, 5 µm.
(C) Representative confocal images of Irgm1+/+ and Irgm1-/- mice BMDMs immunostained with dsRNA (green) and Rig-I (red) antibodies. Scale bar, 8 µm.
(D) Representative confocal images of Irgm1+/+ mice BMDMs immunostained with dsRNA (green) and Mda5 (red) or Rig-I (red) antibodies. Right panel, Scale bar, 8 µm; Left panel, Scale bar, 3 µm.
(E) The qRT-PCR analysis with RNA isolated from Irgm1+/+ and Irgm1-/- mice BMDMs electroporated with RNase III and H (10 unit for 1hr) as indicated. (n = 3 (biological replicates), mean ± SD, *p ≤ 0.05, **p ≤ 0.005, Student's unpaired t-test).
(F) The qRT-PCR analysis with RNA isolated from control and IRGM siRNA knockdown THP-1 cells electroporated with RNase III and H (10 unit for 1hr) as indicated. (n = 3 (technical replicates), mean ± SD, *p ≤ 0.05, **p ≤ 0.005, ***p < 0.0005, Student's unpaired t-test).

Appendix Figure S5

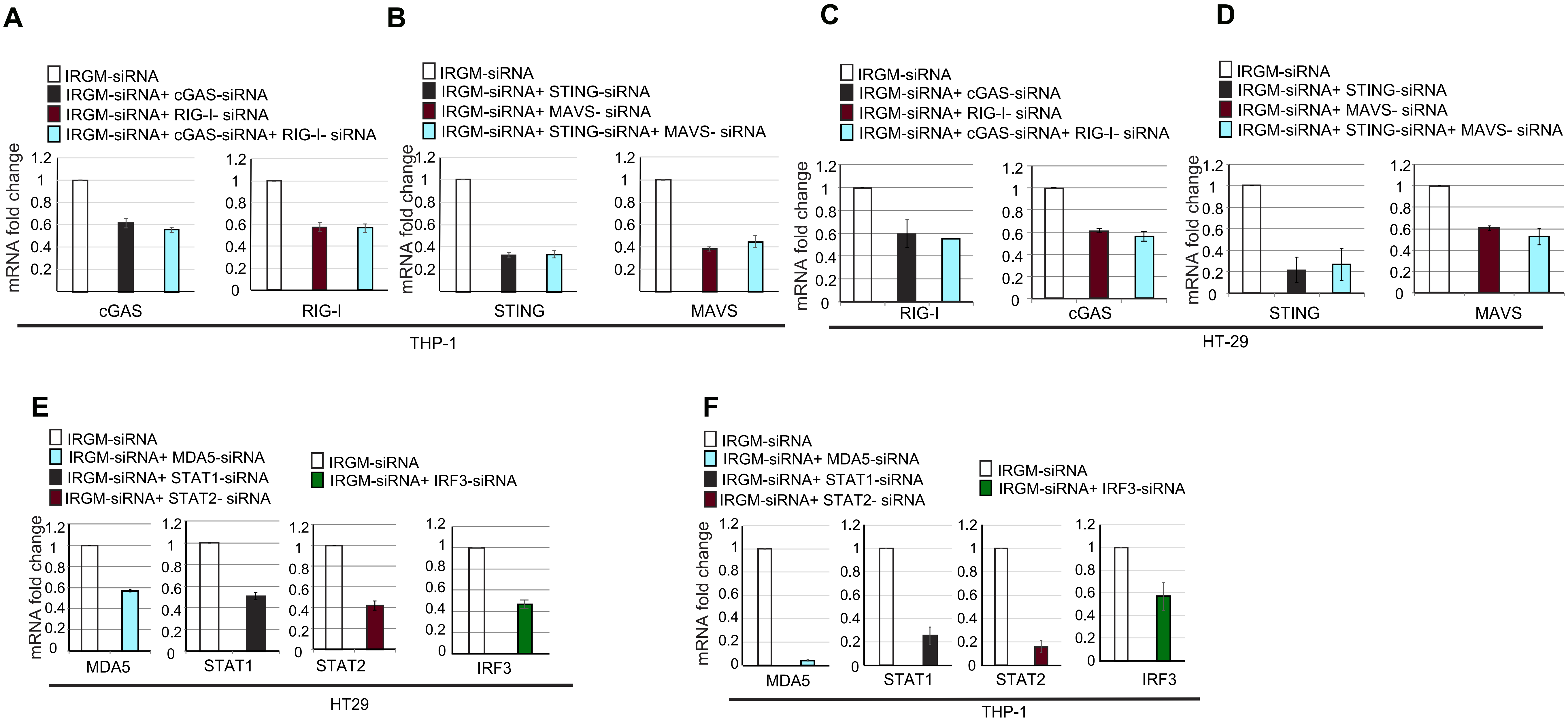

Appendix Figure S5.
(A-B) Control and IRGM knock down THP-1 cells were transfected with indicated siRNA and total RNA was subjected to qRT PCR with (A) cGAS and RIG-I primers (B) STING and-MAVS primers. (n=2, technical replicates, mean ± SD)
(C-D) Control and IRGM knock down HT-29 cells were transfected with indicated siRNA and total RNA was subjected to qRT PCR with (C) RIG-I and cGAS primers (D) STING and MAVS primers. (n=2, technical replicates, mean ± SD)
(E) Control and IRGM knock down HT-29 cells were transfected with indicated siRNA and total RNA was subjected to qRT PCR with MDA5, STAT1, STAT2, and IRF3 primers. (n=2, technical replicates, mean ± SD)
(F) Control and IRGM knock down THP-1 cells were transfected with indicated siRNA and total RNA was subjected to qRT PCR with MDA5, STAT1, STAT2, and IRF3 primers. (n=2, technical replicates, mean ± SD)

Appendix Table S1

Primer sequence for qRT PCR are as follows:

qRT primers:

Mouse Mx1 Forward	AGGCAGTGGTATTGTCACCA
Mouse Mx1 Reverse	AGACTTTGCCTCTCCACTCC
Mouse Mx2 Forward	AGGCAGTGGTATTGTCACCA
Mouse Mx2 Reverse	AGACTTTGCCTCTCCACTCC
Mouse Rig I(Ddx58) Forward	CATTCTCTATGAGTACGTGGGC
Mouse Rig I(Ddx58) Reverse	ACTTGCTATCTCGTGCTCTTC
Mouse cGas Forward	GTGAATCTTCCGGAGCAAAATG
Mouse cGas Reverse	ACATGTGAAAGATGGCAGTTTT
Mouse Mda5 (Ifih1) Forward	TCTGGAGAGTGGAGACGATG
Mouse Mda5 (Ifih1) Reverse	GCCTCCTTGTTGGTGTGATG
Mouse Irf7 Forward	TTGATCCGCATAAGGTGTACG
Mouse Irf7 Reverse	TTCCCTATTTTCCGTGGCTG
Mouse Stat1 Forward	CCAAAGGAAGCACCAGAACC
Mouse Stat 1 Reverse	GGGTGGACTTCAGACACAGA
Mouse Stat 2 Forward	CAAGCTGGACGAGTGGAAG
Mouse Stat 2 Reverse	CGAAGGTGGAACAAGAACTTTG
Mouse Apobec3 Forward	CTGCTAAGCGAGAAAGGCAA
Mouse Apobec3 Reverse	CAGGTGAGGTAGCAGGTGAT
Mouse Oas1a Forward	AGGTGGAGTTTGATGTGCTG
Mouse Oas1a Reverse	GTAGAGAACTCGCCATCCTTC
Mouse Ifn beta Forward	GCACTACAGGCTCCGAGATGAAC
Mouse Ifn beta Reverse	TTGTCGTTGCTTGGTTCTCCTTGT
Mouse Isg15 Forward	GAGAGCAAGCAGCCAGAAG
Mouse Isg15 Reverse	CCCAGGCCATTGCTGCAGGC
Human RIG I(DDX58) Forward	TGTTACACAGCTGACGTAAGAG
Human RIG I(DDX58) Reverse	TGTCGGGCACAGAATATCTTTG
Human MDA5(IFIH1) Forward	TGTCCAAGAGTTAACAGGCTC

Human MDA5(IFIH1) Reverse	GATTTGGCTGAACTGTGGTTG
Human OAS1 Forward	GAAAGGTGCTTCCGAGGTAG
Human OAS1 Reverse	GGACTGAGGAAGACAACCAG
Human MX1 Forward	CCAGTAATGTGGACATCGCC
Human MX1 Reverse	CTTGTCTTCAGTTCCTTTGTCC
Human MX2 Forward	ACTGGCAGAAAGACTTACCAC
Human MX2 Reverse	GGCTCTCCCTTATTTGTCCTTC
Human ISG20 Forward	GAGATCACCGATTACAGAACCC
Human ISG20 Reverse	ACCAGCTTGCCTTTCAGG
Human ISG15 Forward	ACTCATCTTTGCCAGTACAGG
Human ISG15 Reverse	CCAGCATCTTCACCGTCAG
Human STAT1 Forward	GCCTCCATCCTTTGGTACAAC
Human STAT1 Reverse	TTCTGAAAGCTGAGCCCATC
Human STAT2 Forward	ACCCCTCATCCTCAAGACT
Human STAT2 Reverse	AGTGACTCATTGCCTTCCTG
Human TRIM 22 Forward	GCACGCTCATCTCAGATCTC
Human TRIM 22 Reverse	TCAATGTCCAGCTTTCACTCC
Human APOBEC3G Forward	CTGTGTTATGAGGTGGAGCG
Human APOBEC3G Reverse	GAAACCGTGTTTATGTGGAGC
Human p62 Forward	AGCTGCCTTGTACCCACATC
Human p62 Reverse	CAGAGAAGCCCATGGACAG
Human TAX1BP1 Forward	GACCAAAGAAATTGCTGACAAAAC
Human TAX1BP1 Reverse	GCAATTTTCACTTGTTCTTTCCATT
Human NBR1 Forward	AGGTATCCATCAACAGTCAAGG
Human NBR1 Reverse	TCGTTTTGCTCCTACAACTGG
Human NDP52 Forward	TGGGTCTGGATTTTAATTCTTTGC
Human NDP52 Reverse	ATTTCTTGATGGAGAGCGGG
