# Supplementary material for: Autoimmunity gene IRGM suppresses cGAS‐STING and RIG‐I‐MAVS signaling to control interferon response
Source: EMBO Rep. 2020 Jul 27;21(9):e50051. doi: 10.15252/embr.202050051 (PMC7507369; doi:10.15252/embr.202050051)
Supplement: Supplementary file 2 — Expanded View Figures PDF [file EMBR-21-e50051-s002.pdf]

# Expanded View Figures

**Figure EV1. IRGM controls IFN signaling pathways.**

A IRGM knockdown efficiency HT29 stably expressing control shRNA or IRGM shRNA as analyzed using qRT–PCR. Mean ± SD, $n$ = 3 (biological replicates), ***$P$ < 0.0005, Student's unpaired $t$-test.

B The bar graphs represent highly enriched biological pathways upregulated in gene ontology (GO)-based Ingenuity pathway analysis using sets of genes induced (1.5-fold, $P$ < 0.05, Wald test, $n$ = 3) in IRGM shRNA knockdown HT29 cells compared to control shRNA cells.

C qRT–PCR validation of RNA-seq data in control siRNA and IRGM siRNA transfected human PBMC cells. Mean ± SD, $n$ = 3 individual human samples, *$P$ < 0.05, **$P$ < 0.005, Student's unpaired $t$-test.

D Bar graph represents top canonical biological pathways upregulated in GO-based Reactome pathway analysis using sets of genes induced (1.5-fold, $P$ < 0.05, Wald test, $n$ = 3) in *Irgm1*$^{-/-}$ mouse BMDMs compared to *Irgm1*$^{+/+}$ mouse BMDMs. Heatmaps were generated for sentinel interferon-regulated genes (three biological replicates).

E Bar graph represents top canonical biological pathways upregulated in GO-based metascape pathway analysis using sets of genes induced (1.5-fold, $P$ < 0.05, Wald test, $n$ = 3) in *Irgm1* knockout mouse BMDMs compared to wild-type mouse BMDMs.

F Interferome database analysis with sets of genes induced (1.5-fold, $P$ < 0.05, Wald test, $n$ = 3) in BMDMs of *Irgm1*$^{-/-}$ compared to BMDMs of *Irgm1*$^{+/+}$ wild-type mice. The venn diagram depicts the total number of upregulated type I and type II IFN-regulated genes in *Irgm1*$^{-/-}$ mouse BMDMs.

G Diagrammatic representation of MHC-1 antigen processing and presentation pathway.

H Heatmaps of the genes of this pathway differentially expressed (1.5-fold, $P$ < 0.05, Wald test, $n$ = 3) in *Irgm1*$^{+/+}$ and *Irgm1*$^{-/-}$ mouse BMDMs and brain and also control and IRGM knockdown HT29 cells.

I Heatmaps showing genes of complement system pathway upregulated (1.5-fold, $P$ < 0.05, Wald test, $n$ = 3) in the brain of *Irgm1*$^{-/-}$ mice compared to *Irgm1*$^{+/+}$ mice.

J Heatmaps of the TRIM family genes upregulated (1.5-fold, $P$ < 0.05, Wald test, $n$ = 3) in *Irgm1*$^{-/-}$ mouse BMDMs and brain and also IRGM knockdown HT29 cells.

© 2020 The Authors

© 2020 The Authors

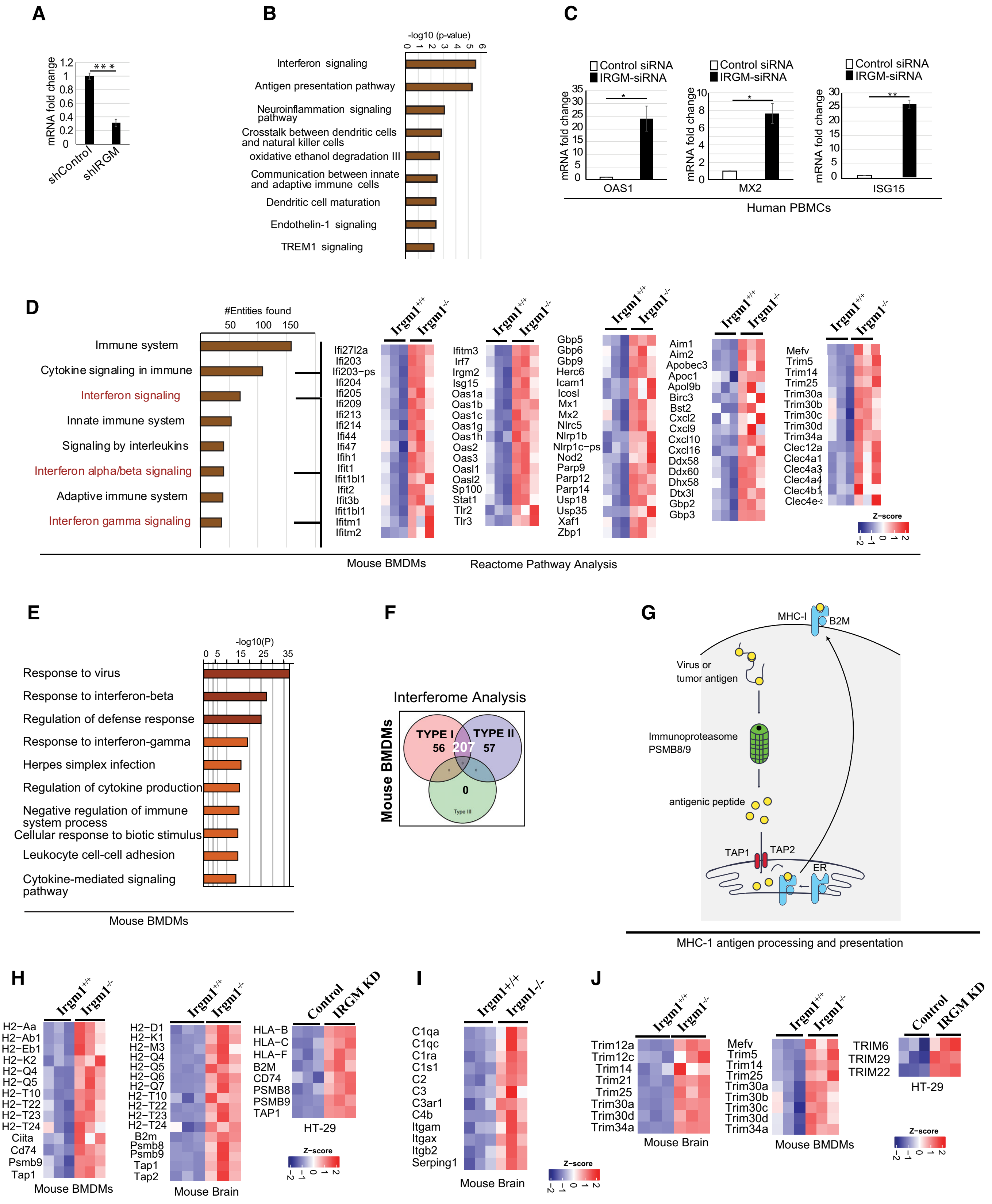


**Figure EV1.**

 © 2020 The Authors

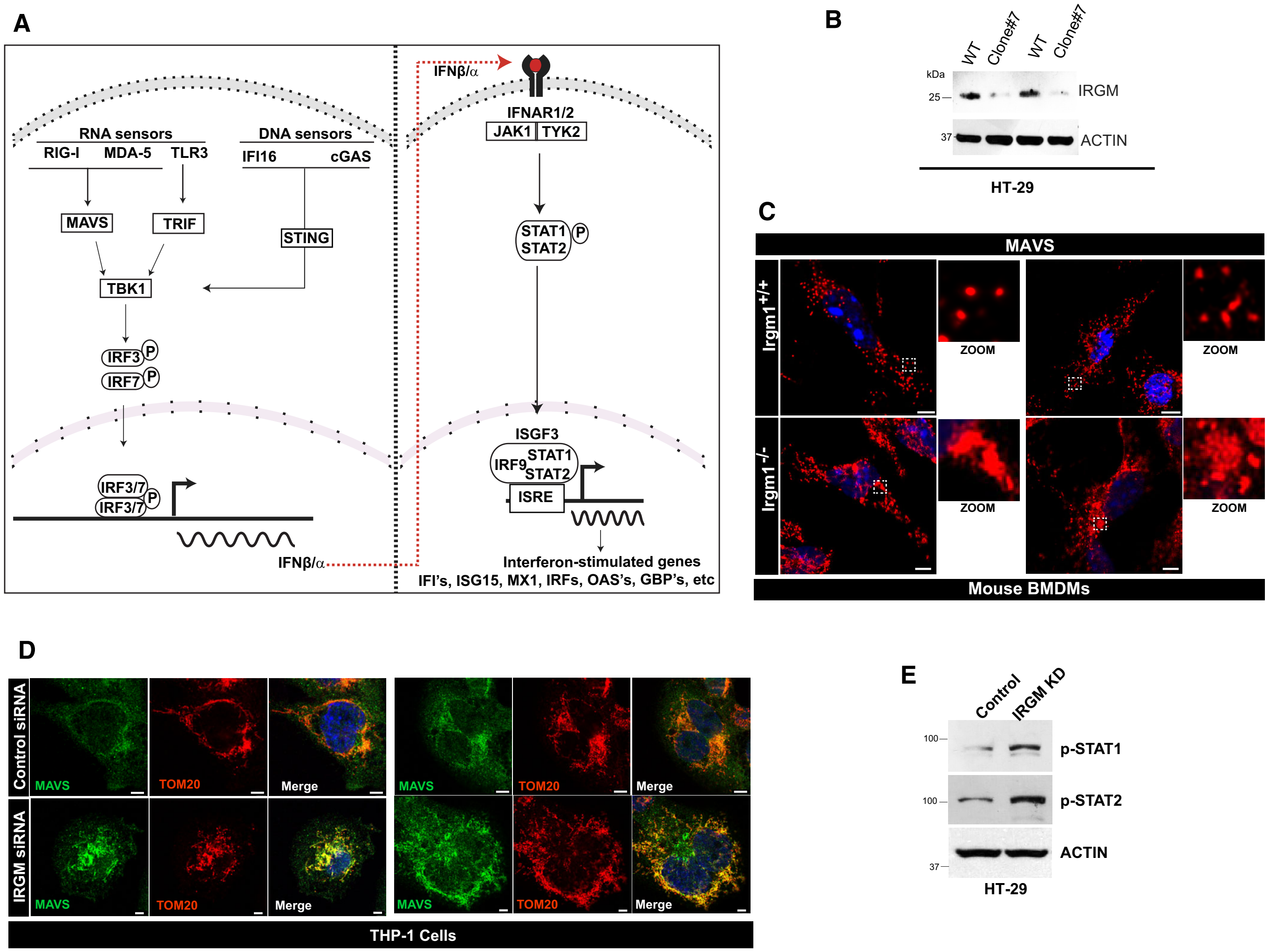


**Figure EV2. IRGM depletion results in constitutively active nucleic acid-sensing and ISG production pathways.**

A The graphical representation of the pathways associated with type I IFN and ISG production.

B Western blot analysis with lysates of wild type and CRISPR-Cas9 clone #7 of HT29 cells. Clone #7 was identified to be a single allele knockout for IRGM.

C Representative confocal images of *Irgm1*$^{+/+}$ and *Irgm1*$^{-/-}$ mouse BMDMs immunostained with MAVS (red) antibody. Scale bar, 5 µm. Zoom panels are digital magnifications.

D Representative confocal images of control siRNA and IRGM siRNA transfected THP-1 cells immunostained with MAVS (green) and TOM20 (red) antibodies. Control cells, scale bar, 5 µm; IRGM siRNA cells, scale bar, 3 µm.

E Western blot analysis to assess activation of p-STAT1 and p-STAT2 in HT29 cells stably expressing control shRNA and IRGM shRNA.

Source data are available online for this figure.

© 2020 The Authors 

**Figure EV3. IRGM interacts and degrades cytoplasmic DNA and RNA sensor proteins.**

- A Co-IP analysis of the interaction between GFP-IRGM and Flag-cGAS, Flag-RIG-I and Flag-TLR3 in HEK293T cell lysates.
- B Western blot analysis with lysates of THP-1 cell transiently transfected (4 h) with control or Flag-IRGM plasmid and probed with indicated antibodies.
- C–E Western blot analysis of HEK293T cell lysates expressing 3X-Flag or Flag-IRGM and (C) Flag-RIG-I or (D) Flag-cGAS or (E) Flag-TLR3.
- F–H HEK293T cells expressing GFP or GFP-IRGM and (F) Flag-TLR3, (G) Flag-cGAS, and (H) Flag-RIG-I were treated with bafilomycin A1 (300 nM, 8 h) or chloroquine (50 μM, 8 h) or MG132 (20 μM, 8 h) and cell lysates were subjected to western blot analysis with the indicated antibodies.
- I The qRT–PCR analysis with RNA isolated form control siRNA, BECLIN1 siRNA or IRGM siRNA transfected THP-1 cells. Mean ± SD, $n = 3$ (biological replicates), *$P < 0.05$, **$P < 0.005$, ***$P < 0.0005$ Student's unpaired *t*-test.
- J The qRT–PCR analysis with RNA isolated form control siRNA, p62 siRNA or IRGM siRNA transfected THP-1 cells. Mean ± SD, $n = 3$ (biological replicates), *$P < 0.05$, **$P < 0.005$, ***$P < 0.0005$ Student's unpaired *t*-test.
- K Co-immunoprecipitation (Co-IP) analysis of the ubiquitination of Flag-RIG-I, Flag-TLR3, or Flag-cGAS in the presence of EGFP or GFP-IRGM and probed with indicated antibodies. IP samples are adjusted according to Input samples.

Data information: #degradation products of full-length GFP-IRGM.

Source data are available online for this figure.

 © 2020 The Authors

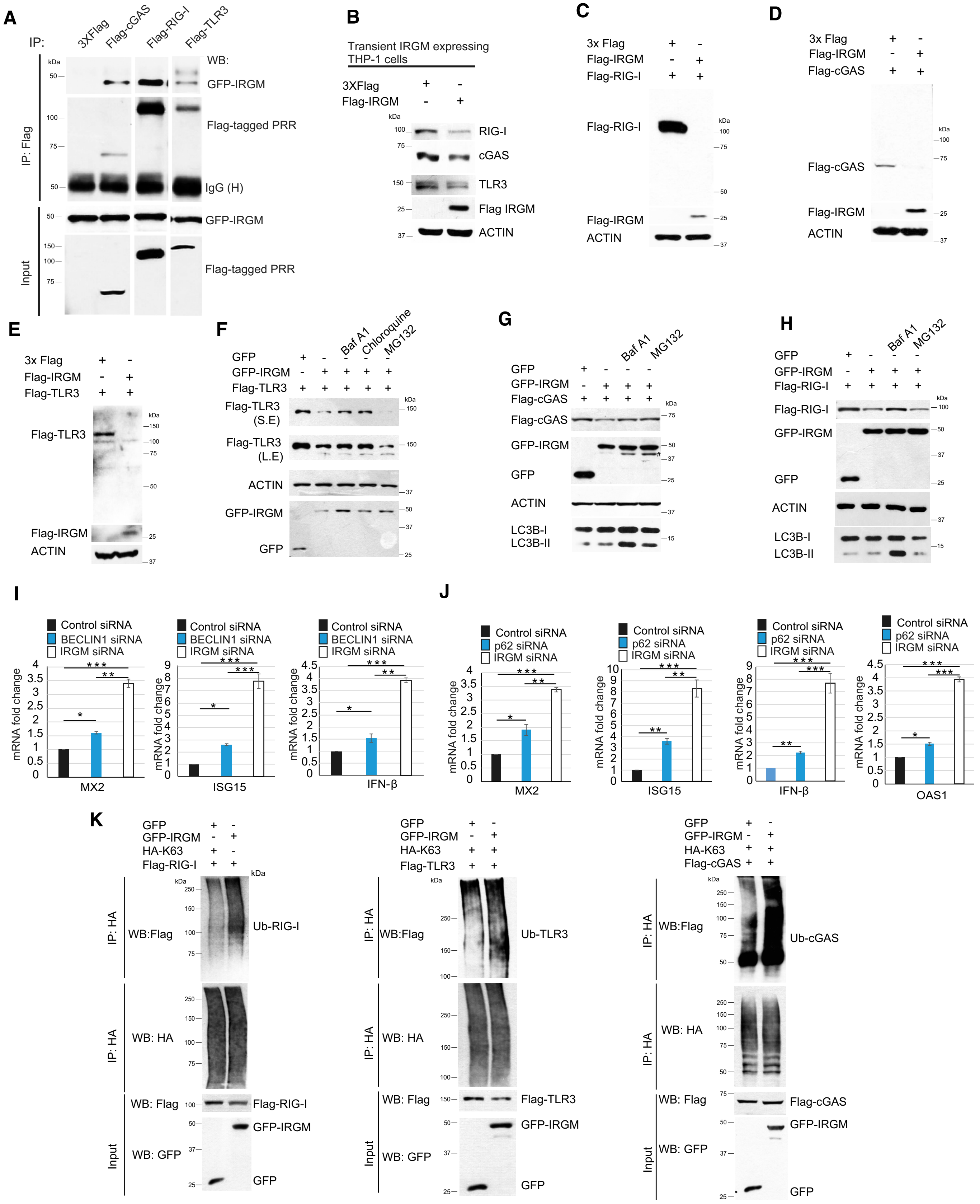
A
IP:
3XFlag
Flag-cGAS
Flag-RIG-I
Flag-TLR3
WB:
GFP-IRGM
Flag-tagged PRR
IgG (H)
IP: Flag
Input
B
Transient IRGM expressing THP-1 cells
3XFlag
Flag-IRGM
RIG-I
cGAS
TLR3
Flag IRGM
ACTIN
C
3x Flag
Flag-IRGM
Flag-RIG-I
ACTIN
D
3x Flag
Flag-IRGM
Flag-cGAS
ACTIN
E
3x Flag
Flag-IRGM
Flag-TLR3
ACTIN
F
GFP
GFP-IRGM
Flag-TLR3
Baf A1
Chloroquine
MG132
Flag-TLR3 (S.E)
Flag-TLR3 (L.E)
ACTIN
GFP-IRGM
GFP
G
GFP
GFP-IRGM
Flag-cGAS
Baf A1
MG132
ACTIN
LC3B-I
LC3B-II
H
GFP
GFP-IRGM
Flag-RIG-I
Baf A1
MG132
ACTIN
LC3B-I
LC3B-II
I
Control siRNA
BECLIN1 siRNA
IRGM siRNA
mRNA fold change
MX2
ISG15
IFN-β
J
Control siRNA
p62 siRNA
IRGM siRNA
mRNA fold change
MX2
ISG15
IFN-β
OAS1
K
GFP
GFP-IRGM
HA-K63
Flag-RIG-I
Flag-TLR3
Flag-cGAS
IP: HA
WB:Flag
WB: HA
Input
WB: Flag
WB: GFP
Ub-RIG-I
Ub-TLR3
Ub-cGAS
Flag-RIG-I
Flag-TLR3
Flag-cGAS
GFP-IRGM
GFP
kDa

**Figure EV3.**

**Figure EV4. IRGM depletion results in defective mitophagy and enhanced cytosolic DAMPs.**

A Representative confocal images of control siRNA and IRGM siRNA transfected THP-1 cells processed for IF analysis with TOM20 antibody. Left panels, scale bar, 5 μm; right panels scale bar, 3 μm. Zoom panels are digital magnifications.

B The graph depicts the percentage of cells with swollen or rounded mitochondria in control siRNA, and IRGM siRNA transfected THP-1 cells. Mean ± SD, $n = 3$ (biological replicates), $^{**}P < 0.005$, Student's unpaired $t$-test.

C Representative confocal images of *Irgm1*$^{-/-}$ mouse BMDMs immunostained with TOM20 (red) and Pink1 (green) antibodies. Right Panel: Co-localization analysis using line intensity profiles. Scale bar, 10 μm. Zoom panels are digital magnifications.

D Representative confocal images of *Irgm1*$^{+/+}$ and *Irgm1*$^{-/-}$ mouse BMDMs transfected with YFP-Parkin (green) and immunostained with Tom20 (red) antibody. Right Panel: Co-localization analysis using line intensity profiles. Scale bar, 5 μm.

E Western blot analysis of the mitochondrial fraction of control siRNA and IRGM siRNA transfected THP-1 cells untreated or treated with bafilomycin A1 (300 nM, 4 h), probed with indicated antibodies.

F Flow cytometry analysis of control siRNA and IRGM siRNA transfected human PBMCs from two donors stained with CMXRos red dye (10 nM, 30 min).

G Representative flow cytometry analysis of control and IRGM siRNA transfected human PBMCs from two donors stained with CellRox green dye (1 μM, 30 min).

H Representative confocal images of *Irgm1*$^{+/+}$ and *Irgm1*$^{-/-}$ mouse BMDMs immunostained with dsDNA (green) and Cytochrome-C (red) antibodies. Zoom panels show extracellular DNA in the cytoplasm. Dashed lines indicate the periphery of the cells. Upper panels, scale bar, 5 μm; lower panels, scale bar, 10 μm. Zoom panels are digital magnifications.

I Representative confocal images of control siRNA and IRGM siRNA transfected THP-1 cells immunostained with dsDNA (green) and TOM20 (red) antibodies. Scale bar, 3 μm.

J Representative confocal images of control siRNA and IRGM siRNA transfected HT-29 cells immunostained with dsDNA (green) antibodies and DAPI (blue) staining. Scale bar, 8 μm.

K qRT–PCR analysis with RNA isolated form control siRNA, PARKIN siRNA or IRGM siRNA transfected THP-1 cells. Mean ± SD, $n = 3$ (biological replicates), $^{*}P < 0.05$, $^{**}P < 0.005$, $^{***}P < 0.0005$ Student's unpaired $t$-test.

L Western blot analysis of lysates prepared from control siRNA and PARKIN siRNA transfected THP-1 cells, probed with indicated antibodies.

M qRT–PCR analysis with RNA isolated form control siRNA or IRGM siRNA transfected THP-1 cells and treated with MDIVI-1 (50 μg/ml, 1 h). Mean ± SD, $n = 3$ (biological replicates), $^{*}P < 0.05$, $^{**}P < 0.005$, $^{***}P < 0.0005$, $^{\#}$insignificant. Student's unpaired $t$-test.

Data information: The microscopic scales are as depicted in the images.

Source data are available online for this figure.


© 2020 The Authors

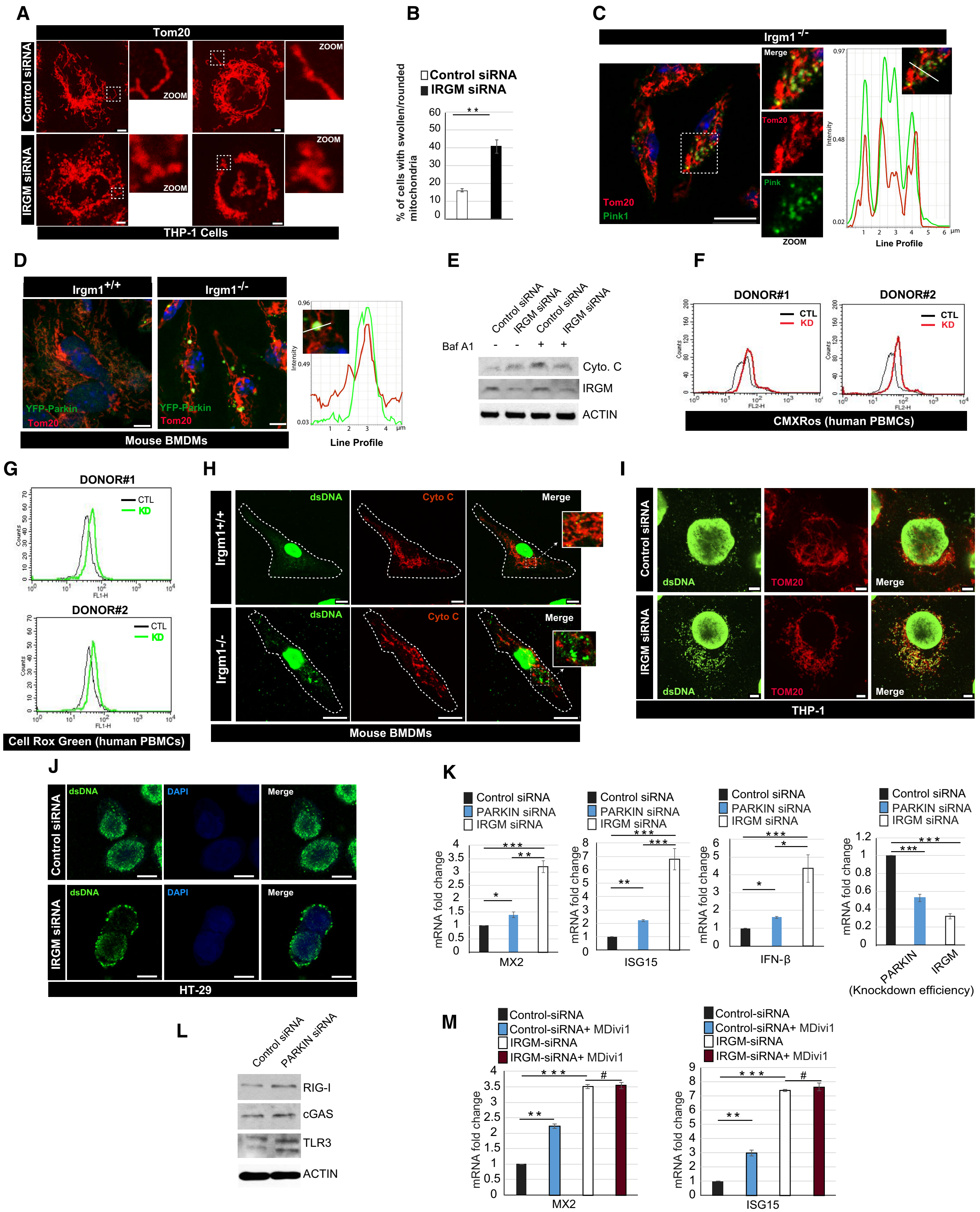
A
Tom20
Control siRNA
IRGM siRNA
ZOOM
THP-1 Cells
B
Control siRNA
IRGM siRNA
% of cells with swollen/rounded mitochondria
C
Irgm1 -/-
Merge
Tom20
Pink1
ZOOM
Line Profile
D
Irgm1 +/+
Irgm1 -/-
YFP-Parkin
Tom20
Mouse BMDMs
Line Profile
E
Control siRNA
IRGM siRNA
Baf A1
Cyto. C
IRGM
ACTIN
F
DONOR#1
DONOR#2
CTL
KD
CMXRos (human PBMCs)
G
Cell Rox Green (human PBMCs)
H
dsDNA
Cyto C
Merge
Irgm1+/+
Irgm1-/-
Mouse BMDMs
I
TOM20
THP-1
J
DAPI
HT-29
K
PARKIN siRNA
mRNA fold change
MX2
ISG15
IFN-β
PARKIN
IRGM
(Knockdown efficiency)
L
RIG-I
cGAS
TLR3
M
Control-siRNA
Control-siRNA+ MDivi1
IRGM-siRNA
IRGM-siRNA+ MDivi1

**Figure EV4.**

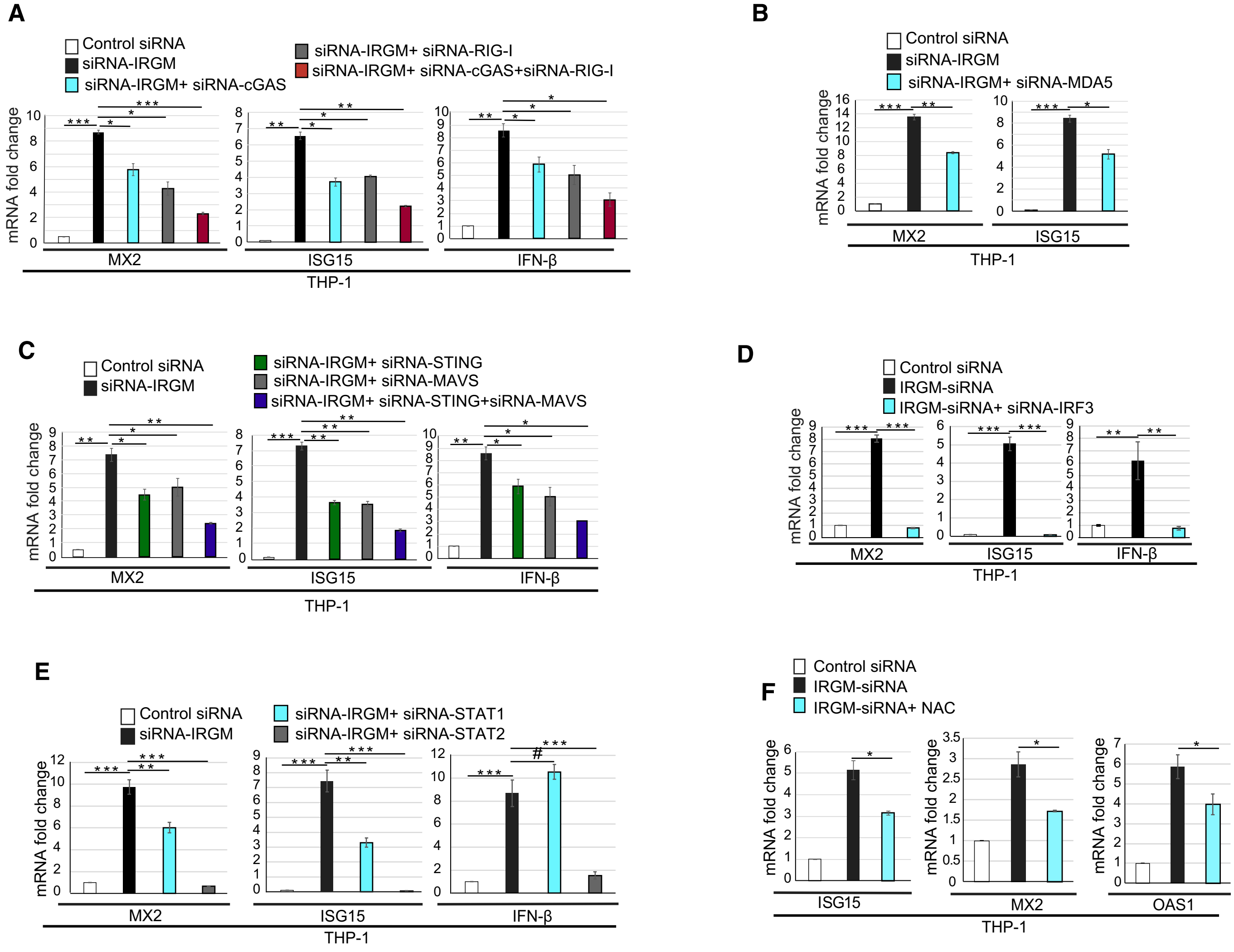


**Figure EV5. The cGAS-STING and RIG-I-MAVS signaling pathways synergistically activate type 1 IFN response in IRGM-depleted cells.**

A qRT–PCR analysis with total RNA isolated from THP-1 cells transfected with control siRNA or IRGM siRNA or doubly transfected with IRGM siRNA and cGAS siRNA or IRGM siRNA and RIG-I siRNA or transfected with three siRNAs as indicated. Mean ± SD, $n$ = 3 (biological replicates), *$P < 0.05$, **$P < 0.005$, ***$P < 0.0005$, Student's unpaired $t$-test

B qRT–PCR analysis with total RNA isolated from THP-1 cells transfected with control siRNA or IRGM siRNA or doubly transfected with IRGM siRNA and MDA5 siRNA as indicated. Mean ± SD, $n$ = 3 (biological replicates), *$P < 0.05$, **$P < 0.005$, ***$P < 0.0005$, Student's unpaired $t$-test.

C qRT–PCR analysis with total RNA isolated from THP-1 cells transfected with control siRNA or IRGM siRNA or doubly transfected with IRGM siRNA and STING siRNA or IRGM siRNA and MAVS siRNA or transfected with all the three siRNAs as indicated. Mean ± SD, $n$ = 3 (biological replicates), *$P < 0.05$, **$P < 0.005$, ***$P < 0.0005$, Student's unpaired $t$-test.

D qRT–PCR analysis with total RNA isolated from THP-1 cells transfected with control siRNA or IRGM siRNA or doubly transfected with IRGM siRNA and IRF3 siRNA as indicated. Mean ± SD, $n$ = 3 (biological replicates), **$P < 0.005$, ***$P < 0.0005$, Student's unpaired $t$-test.

E qRT–PCR analysis with total RNA isolated from THP-1 cells transfected with control siRNA or IRGM siRNA or doubly transfected with IRGM siRNA and STAT1 siRNA or IRGM siRNA and STAT2 siRNA as indicated. Mean ± SD, $n$ = 3 (biological replicates), **$P < 0.005$, ***$P < 0.0005$, $^{\#}P$ = non-significant, Student's unpaired $t$-test.

F qRT–PCR analysis with RNA isolated for control siRNA or IRGM siRNA transfected THP-1 cells untreated or treated with $N$-acetyl-L-cysteine (NAC, 1 mM, 2 h). Mean ± SD, $n$ = 3 (biological replicates), *$P < 0.05$, Student's unpaired $t$-test.

 © 2020 The Authors
