# Supplementary figures and images for: Autoimmunity gene IRGM suppresses cGAS‐STING and RIG‐I‐MAVS signaling to control interferon response
Source: EMBO Rep. 2020 Jul 27;21(9):e50051. doi: 10.15252/embr.202050051 (PMC7507369; doi:10.15252/embr.202050051)

Appendix Figure S1

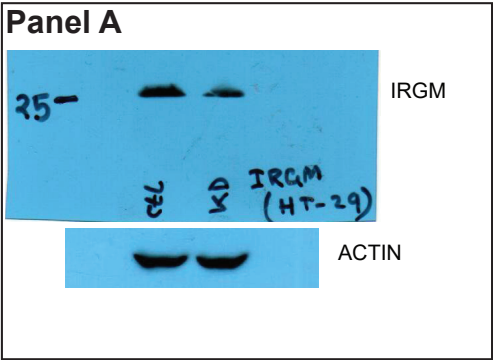

Supplement: Supplementary file 5 — Source Data for Expanded View and Appendix [file EMBR-21-e50051-s009.zip › EV_and_Appendix_Figure_Source_Data/Appendix_FigureS1_Source_Data.pdf]

**Figure EV3**

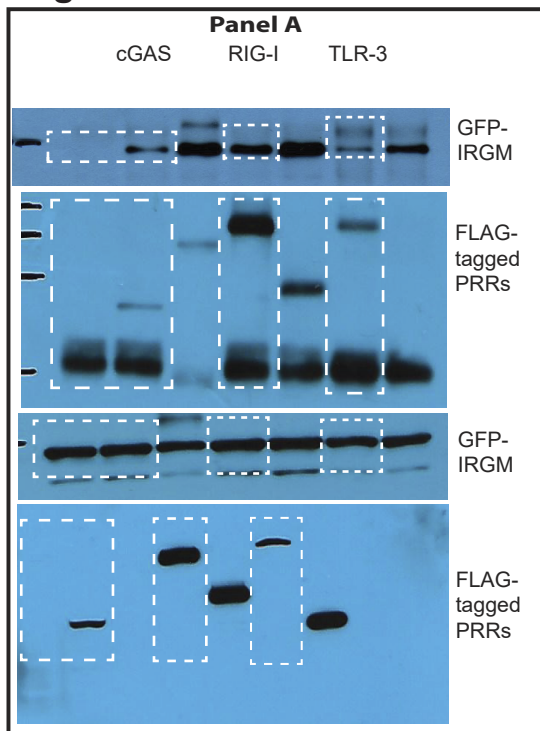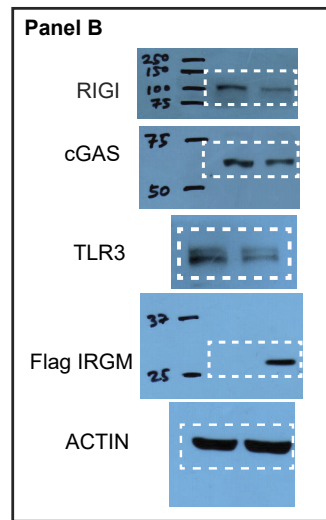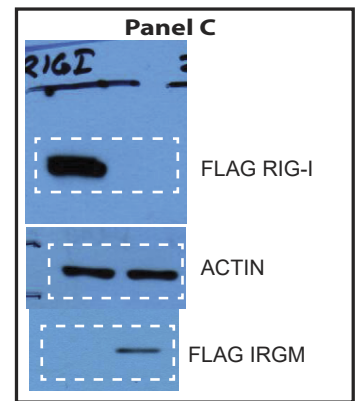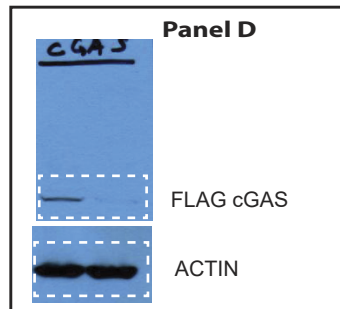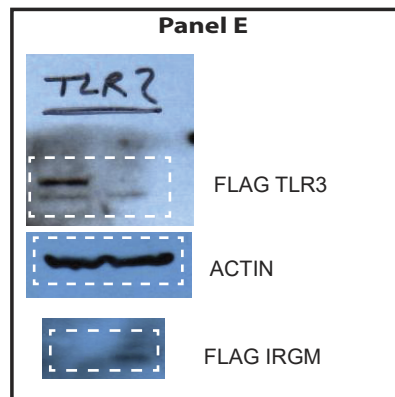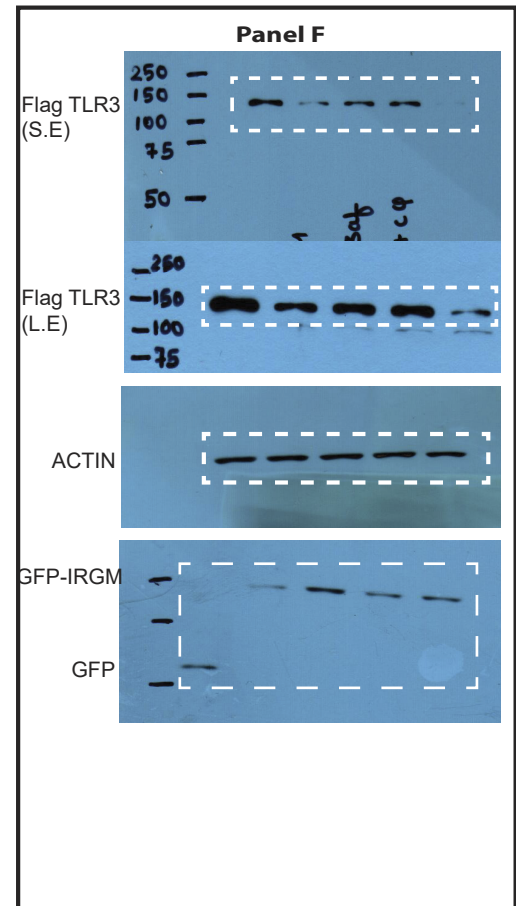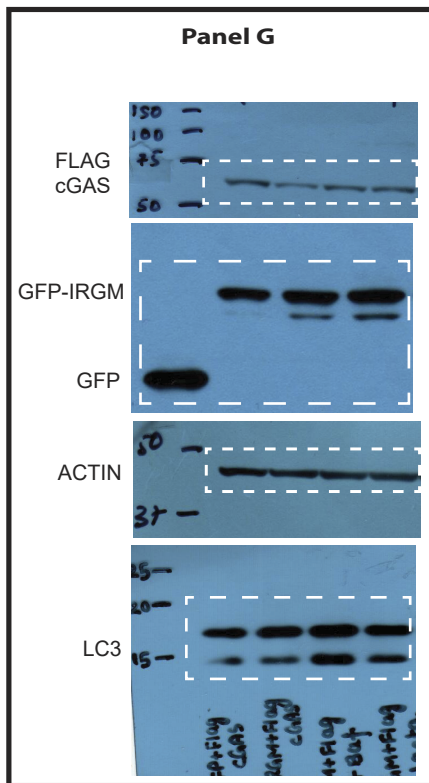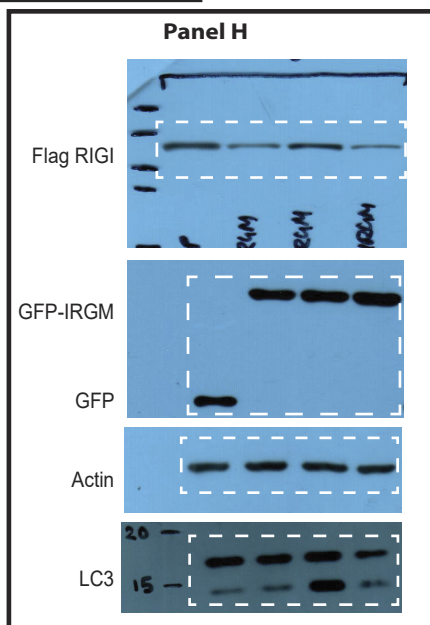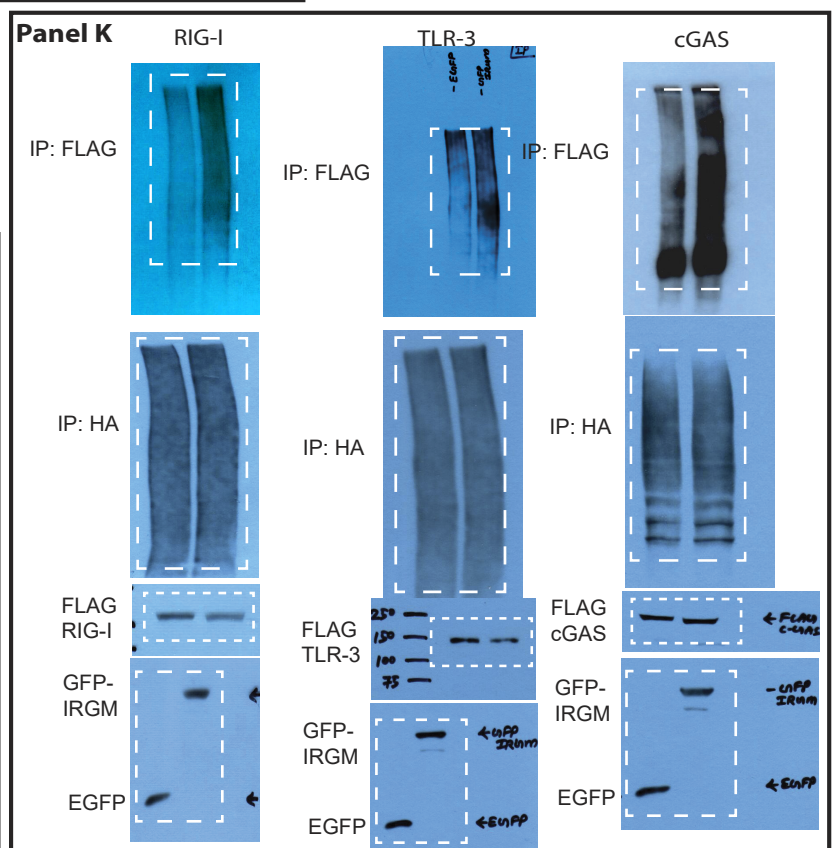

Supplement: Supplementary file 5 — Source Data for Expanded View and Appendix [file EMBR-21-e50051-s009.zip › EV_and_Appendix_Figure_Source_Data/EMBOR-2020-50051V4-Figure_EV3_Source_Data-sd.pdf]

Figure EV2

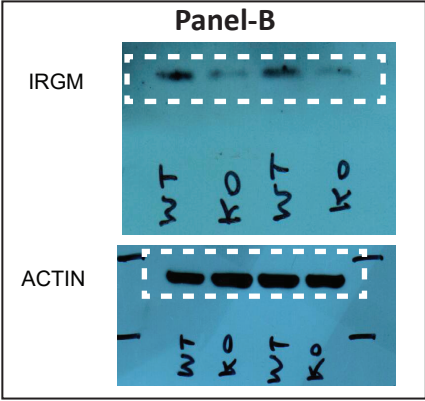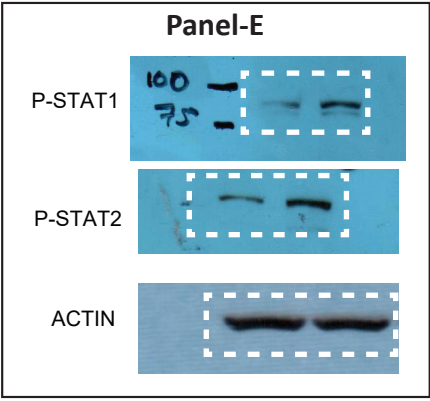

Supplement: Supplementary file 5 — Source Data for Expanded View and Appendix [file EMBR-21-e50051-s009.zip › EV_and_Appendix_Figure_Source_Data/EMBOR-2020-50051V4-Figure_EV2_Source_Data-sd.pdf]

Appendix Figure S2

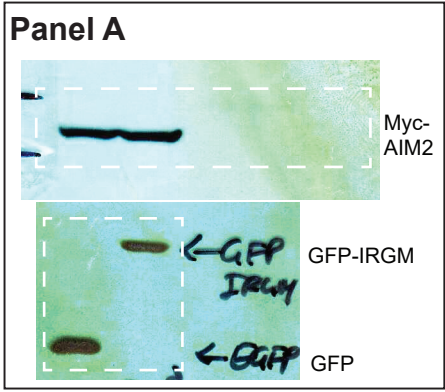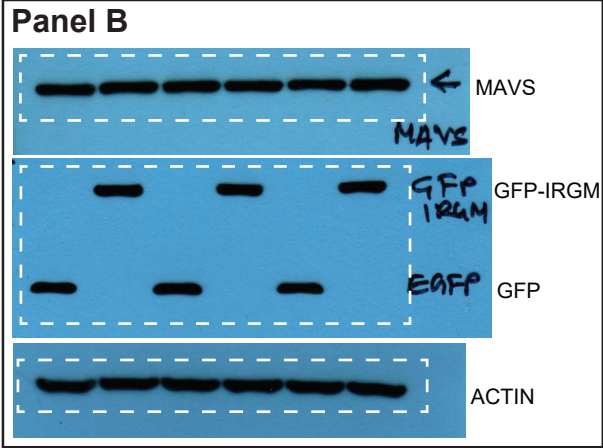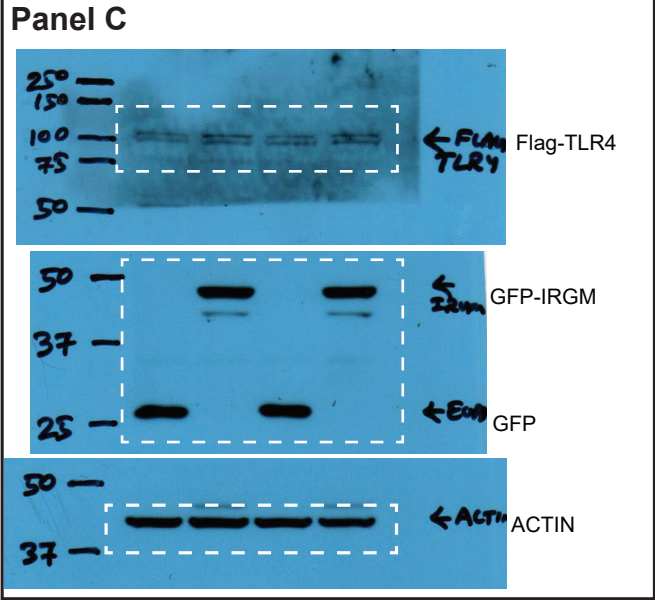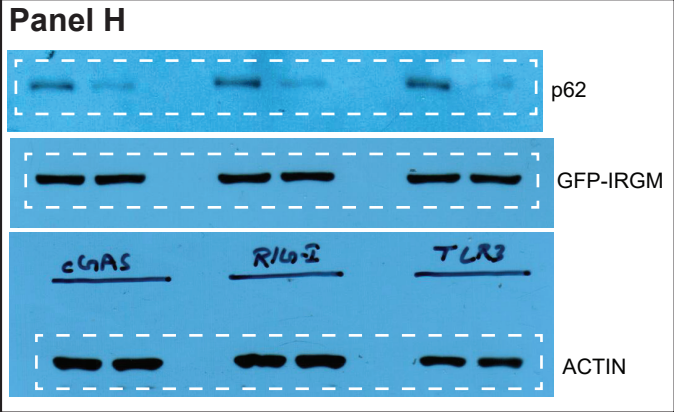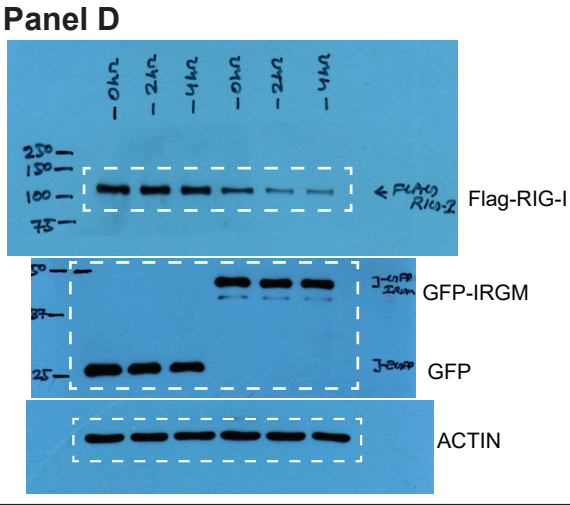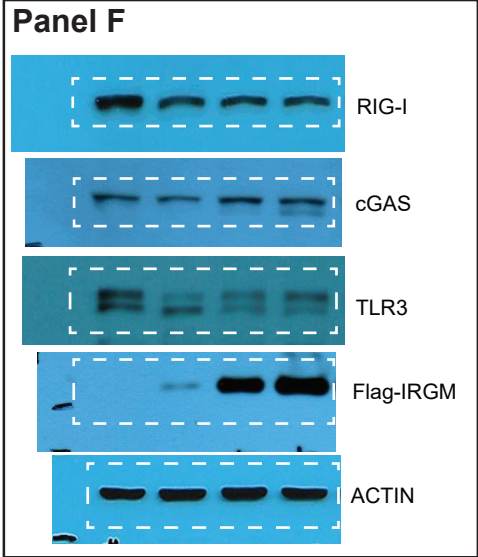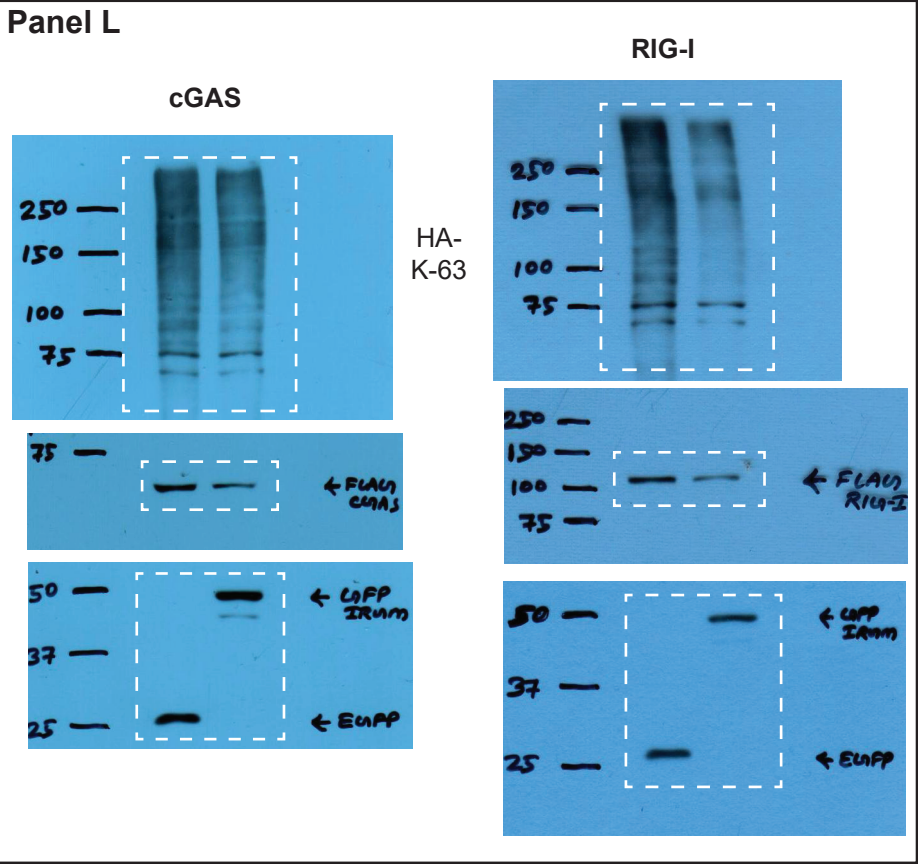

Supplement: Supplementary file 5 — Source Data for Expanded View and Appendix [file EMBR-21-e50051-s009.zip › EV_and_Appendix_Figure_Source_Data/Appendix_FigureS2_Source_Data.pdf]

Appendix Figure S3

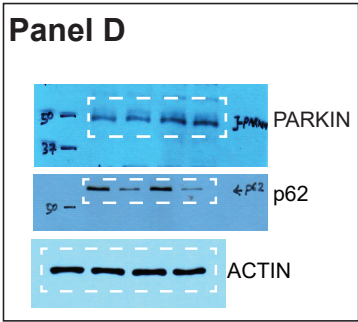

Supplement: Supplementary file 5 — Source Data for Expanded View and Appendix [file EMBR-21-e50051-s009.zip › EV_and_Appendix_Figure_Source_Data/Appendix_FigureS3_Source_Data.pdf]

Figure EV4

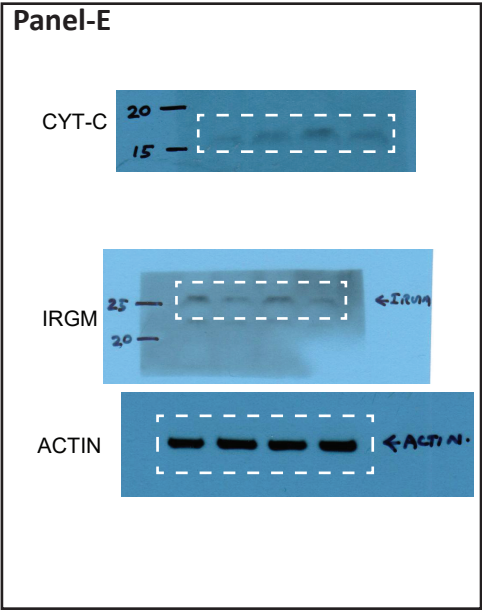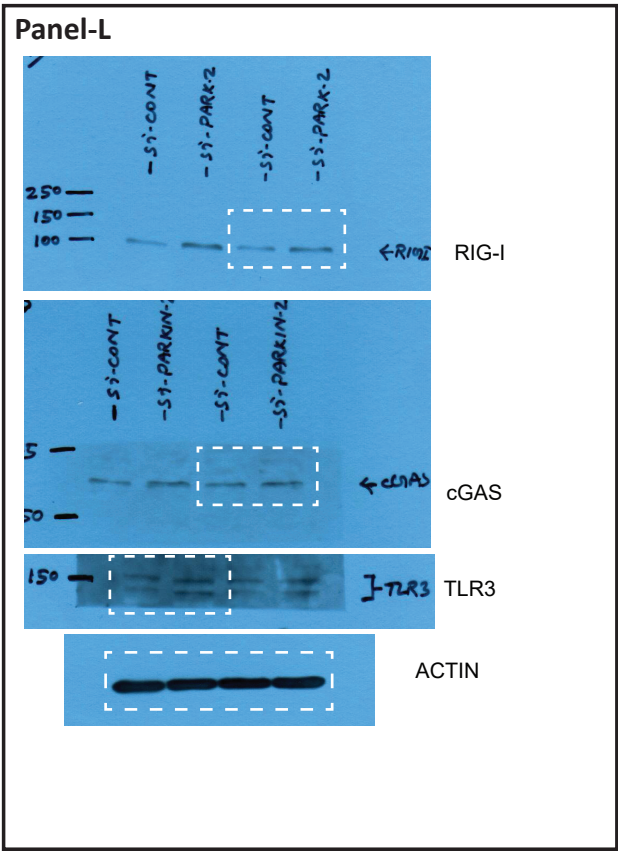

Supplement: Supplementary file 5 — Source Data for Expanded View and Appendix [file EMBR-21-e50051-s009.zip › EV_and_Appendix_Figure_Source_Data/EMBOR-2020-50051V4-Figure_EV4_Source_Data-sd.pdf]

Figure 2

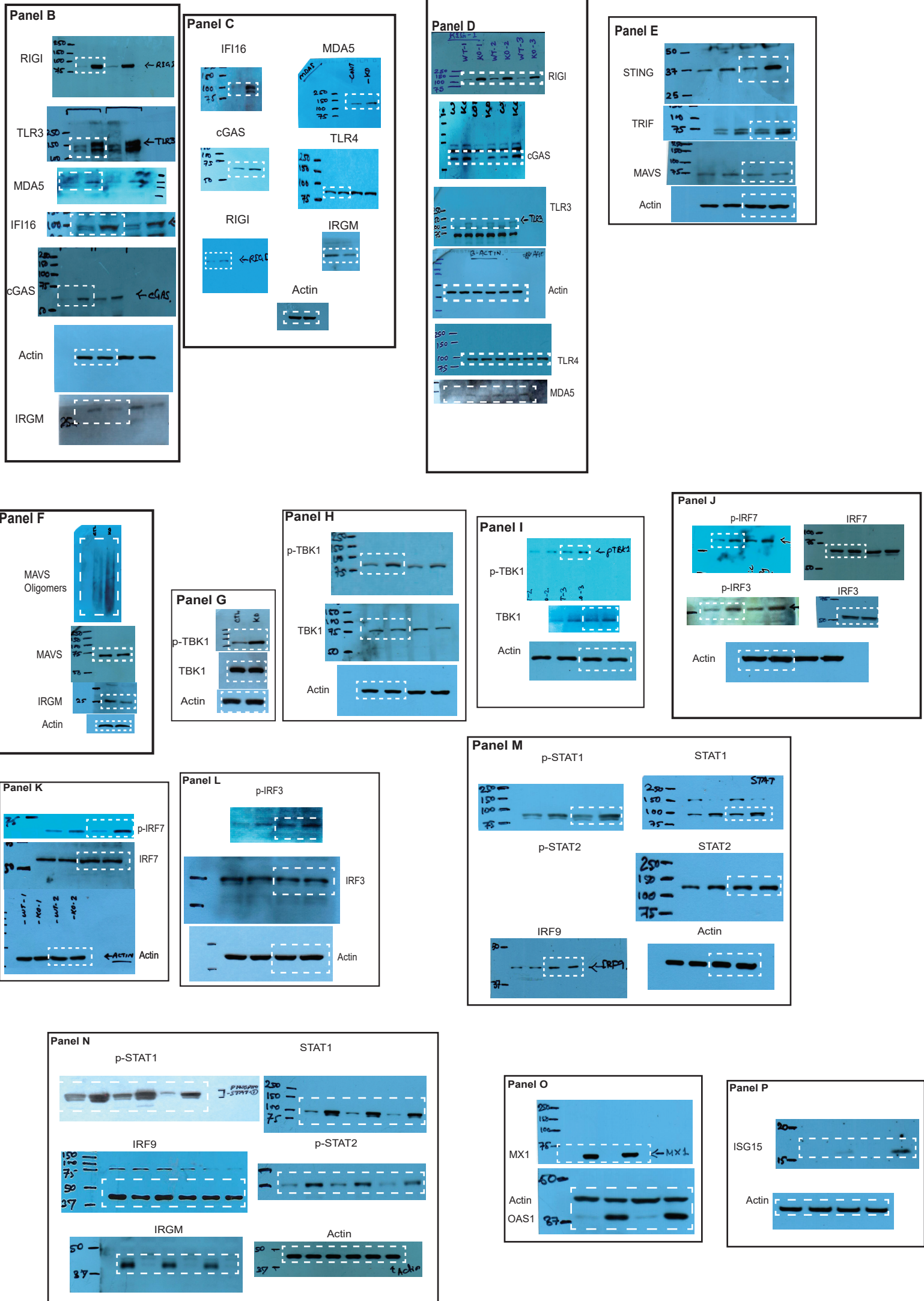

Supplement: Supplementary file 7 — Source Data for Figure 2 [file EMBR-21-e50051-s005.pdf]

Figure 3

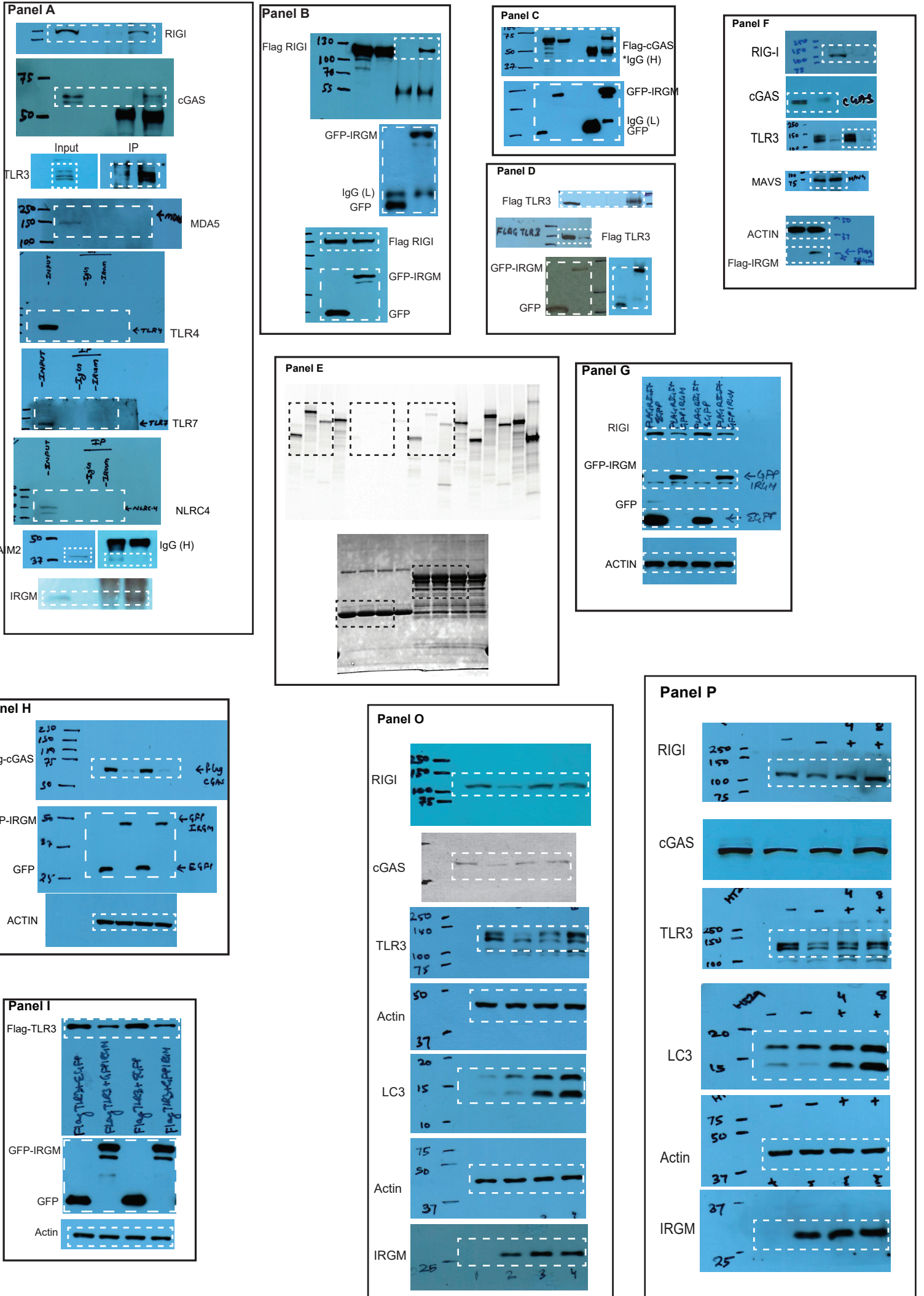

Supplement: Supplementary file 8 — Source Data for Figure 3 [file EMBR-21-e50051-s006.pdf]

Figure 4

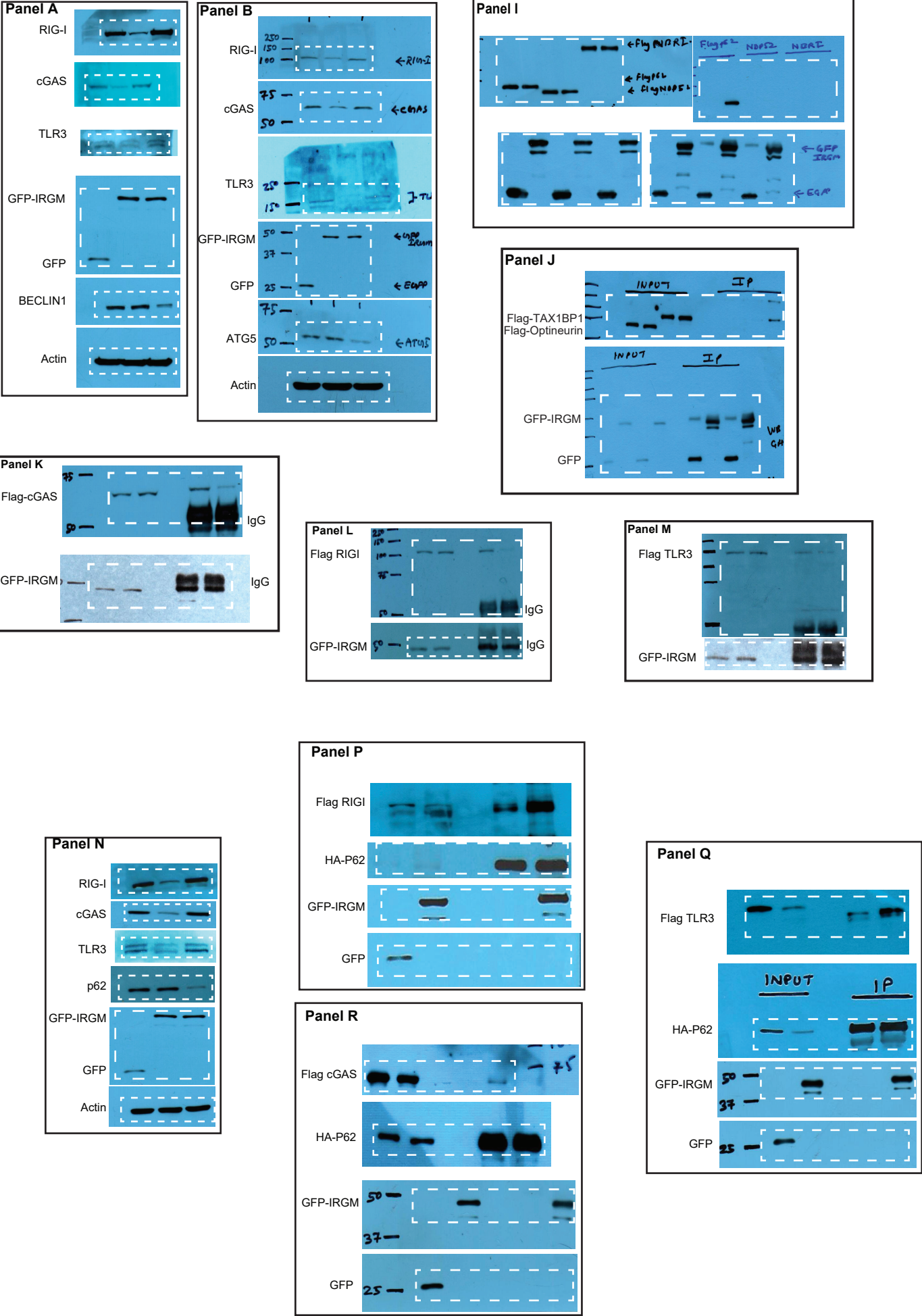

Supplement: Supplementary file 9 — Source Data for Figure 4 [file EMBR-21-e50051-s007.pdf]

Figure 5

Panel F

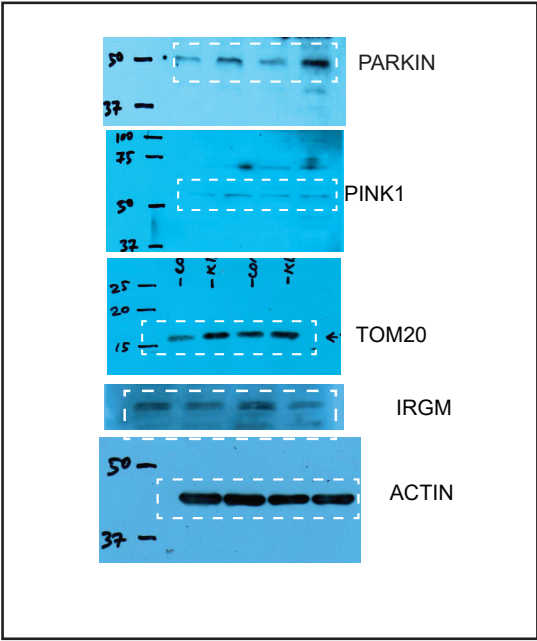

Panel G

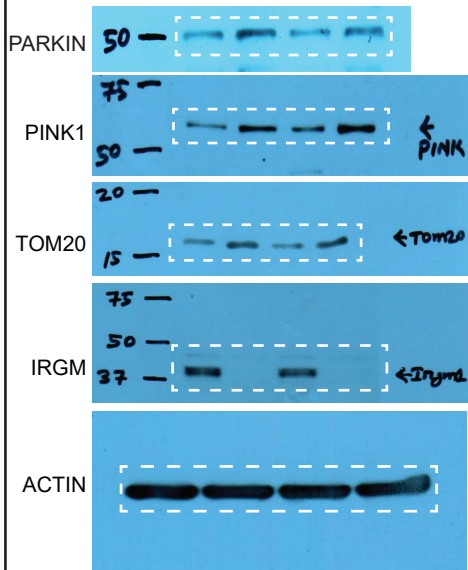

Panel H

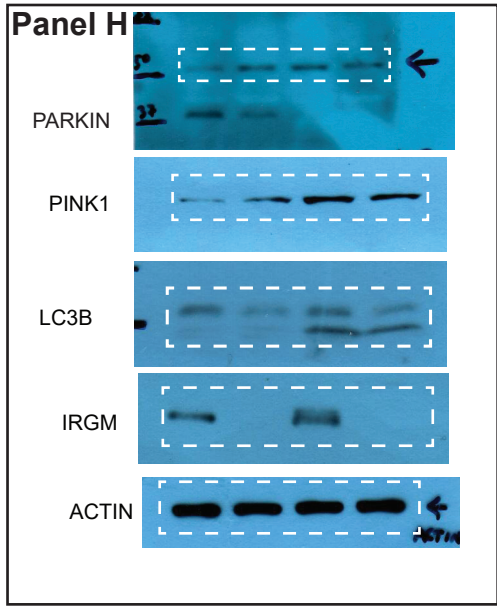

PANEL I

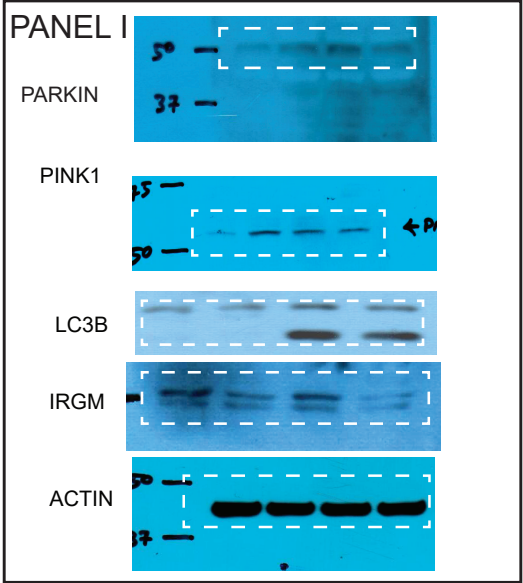

Supplement: Supplementary file 10 — Source Data for Figure 5 [file EMBR-21-e50051-s008.pdf]
